# *Suxiao Jiuxin* Pill Induces Potent Relaxation and Inhibition on Contraction in Human Artery and the Mechanism

**DOI:** 10.1155/2014/956924

**Published:** 2014-04-07

**Authors:** Xiao-Yan Bai, Ping Zhang, Qin Yang, Xiao-Cheng Liu, Jun Wang, Yong-Ling Tong, Song-Jin Xiong, Li-Hua Liu, Lei Wang, Guo-Wei He

**Affiliations:** ^1^TEDA International Cardiovascular Hospital & The Affiliated Hospital of Hangzhou Normal University, Tianjin & Hangzhou, China; ^2^Tianjin Zhongxin Pharmaceutical Group Co., Ltd. No. 6, Traditional Chinese Medicine Factory, Tianjin, China; ^3^The Chinese University of Hong Kong, Hong Kong; ^4^TEDA International Cardiovascular Hospital, Tianjin, China; ^5^TEDA School of Biological Sciences and Biotechnology, Nankai University, Tianjin, China; ^6^Department of Surgery, Oregon Health and Science University, Portland, OR, USA

## Abstract

*Suxiao Jiuxin* Pill, a compound Chinese traditional medicine with main components of tetramethylpyrazine and borneol, is widely used for antiangina treatment in China but its pharmacological effect on human blood vessels is unknown. We investigated the effect and possible mechanism of SJP in the human internal mammary artery (IMA, *n* = 78) taken from patients undergoing coronary surgery. SJP caused full relaxation in KCl- (99.4 ± 10.5%, *n* = 6) and U46619- (99.9 ± 5.6%, *n* = 6) contracted IMA. Pretreatment of IMA with plasma concentrations of SJP (1 mg/mL), calculated from the plasma concentration of its major component borneol, significantly depressed the maximal contraction to KCl (from 35.8 ± 6.0 mN to 12.6 ± 5.6 mN, *P* = 0.03) and U46619 (from 19.4 ± 2.9 mN to 5.7 ± 2.4 mN, *P* = 0.007) while SJP at 10 mg/mL abolished the subsequent contraction. Endothelium denudation and inhibition of eNOS significantly altered the SJP-induced relaxation without changes of eNOS expression. We conclude that SJP has a potent inhibitory effect on the vasoconstriction mediated by a variety of vasoconstrictors in human arteries. The vasorelaxation involves both endothelium-dependent and -independent mechanisms. Thus, the effect of SJP on human arteries demonstrated in this study may prove to be particularly important in vasorelaxing therapy in cardiovascular disease.

## 1. Introduction

Cardiovascular disease (CVD), particularly coronary heart disease (CHD), remains a major cause of mortality, morbidity, and disability in the US and other Westernized societies [1] as well as in low-income and middle-income countries [2]. A Chinese traditional medicine pill named* Suxiao Jiuxin-Wan* (*Suxiao Jiuxin* Pill) has been widely used for treatment of cardiovascular diseases in China on its own or in conjunction with conventional western treatments for angina pectoris, such as with beta blockers, calcium channel blockers, and nitrates (glyceryl trinitrate, GTN) [3, 4]. In fact,* Suxiao Jiuxin* Pill has been added on the national essential drug list of China for the treatment of cardiocerebral vascular diseases [5, 6].* Suxiao Jiuxin* Pill has been shown to cause remission of angina pectoris [7], improve anginal symptoms, and reduce the use of GTN—a drug used to quickly relieve angina symptoms [8].

*Suxiao Jiuxin* Pill is a compound Chinese traditional medicine. The main components of* Suxiao Jiuxin* Pill include the major components extracted from Ligusticum Chuanxiong Hort, that is, tetramethylpyrazine (TMP, Ligustrazine, or* Chuan-Xiong-Qin*), and borneol (Borneolum Syntheticum or* Bing Pian*). The average recoveries of TMP and borneol were 87.38% and 97.32% [9]. In addition, five metal elements including calcium, magnesium, iron, copper, and zinc in* Suxiao Jiuxin* Pill are comparatively rich [10].

TMP is the biologically active ingredient isolated from a popular Chinese medicinal plant Ligusticum Chuanxiong Hort (Szechwan Lovage Rhizome, or* Chuan-Xiong*) that is a perennial Chinese herb, with the effect of blood circulation of* Qi*, chills and pain relief, and gloomy dampness in traditional Chinese medicine for thousands of years [11].

TMP has been synthesized and used widely in oriental medicine since the 1970s to effectively treat several cardiovascular diseases including ischemic heart disease, cerebrovascular and thrombotic vascular diseases [12]. The pharmacological effect of TMP is well established including alleviating kidney and brain damage induced by ischemia perfusion in rats via scavenging oxygen-free radicals [13, 14], significantly suppressing oxidative stress, and attenuating cell death in neuronal cultures induced by glutamate analogue and iron-mediated oxidative stress [15–18].

Borneol is a bicyclic organic compound and a terpene. The hydroxyl group in this compound is placed in an endoposition. Borneol is easily oxidized to the ketone (camphor). One historical name for borneol is Borneo camphor which explains the name [19].* Cinnamomum camphora* is native to south of the Yangtze River of China, Taiwan, Japan, Korea, and Vietnam and has been introduced to many other countries [20].

Borneol is used in traditional Chinese medicine as moxa. In modern era, it is one of the active compounds of the Heart-Protecting Musk Pellets [21]. An early description is found in the* Bencao Gangmu* (*Compendium of Materia Medica* or* Ben-Cao-Gang-Mu*) published in 1590 by* Li Shi-Zhen*.

Despite the above-mentioned and well-known effect of these compounds, the pharmacological effect of* Suxiao Jiuxin* Pill on the human vessels is not reported and this would provide important information as to the clinical use of* Suxiao Jiuxin* Pill in patients with coronary artery disease.

It is well known now that coronary artery bypass grafting (CABG) surgery is the effective therapeutic method for coronary artery disease and the internal mammary artery (IMA) is the most commonly used arterial graft due to its superior graft patency and increased long-term survival [22–25]. Graft spasm of the IMA is a long recognized problem [23, 26–28]. There have been extensive investigations on the antispastic therapy in the past decades to solve this problem and such efforts have greatly reduced the problem of spasm in arterial grafts [22, 29–32]. However, spasm of the IMA may occur even in the current practice as recently reported [33, 34]. The mechanism of graft vasospasm is a complex physiological and pathophysiological issue and may involve mechanical, physical, and pharmacological stimulation. Therefore, reversal and prevention of graft vasospasm would be a challenging task for a long time.

Further, as is well known now, the vascular endothelium plays a crucial role in the maintenance of vascular tone and nitric oxide (NO) produced by endothelial NO synthase (eNOS) is required for normal vascular function. Alteration of NO synthesis has been recognized as a common mechanism by which several cardiovascular risk factors mediate certain deleterious effect on the endothelium.

Taken together, the present study was designed to investigate the vasorelaxation induced by* Suxiao Jiuxin* Pill and its inhibitory effects on vasoconstriction mediated by various vasoconstrictors as well as protein expressions of eNOS in the human IMA.

## 2. Material and Methods

### 2.1. Vessels Preparation

Human IMA (*n* = 78, from 30 patients) segments were obtained from patients undergoing CABG. Approval to use discarded IMA tissue was given by the Institutional Review Board of TEDA International Cardiovascular Hospital with patient consent. The details of the procedure were reported before [29, 35–38]. During CABG, using IMA grafts, the discarded distal end was carefully removed and immediately placed in a container with oxygenated physiological salt solution maintained at 4°C and transferred to the laboratory within 5–10 minutes. After the adherent connective tissue was carefully disposed, the IMA was precisely cut into 3 mm long segments. The Krebs solution was composed of (in mM) Na^+^ 144, K^+^ 5.9, Ca^2+^ 2.5, Mg^2+^ 1.2, Cl^−^ 128.7, HCO_3_^−^ 25, SO_4_^2−^ 1.2, H_2_PO_4_^−^ 1.2, and glucose 11, which was aerated with a gas mixture of 95% O_2_ and 5% CO_2_ at 37°C ± 0.1°C during the period of experiment.

### 2.2. Organ Bath Techniques

Human IMA ring segments were suspended on wire hooks in a 10 mL bath on a myograph modified for large vessel studies (Model 610M, DMT Company, Denmark). Each ring segment unstretched on the wire hooks was equilibrated in Krebs solution for at least 30 minutes. 

*Normalization*. The organ bath technique described previously in detail was used in this study [29, 39, 40]. Briefly, each ring segment was stretched up in progressive steps every minute to determine the individual length-tension curve. A computer iterative fitting program (Myodaq and Myodata, version 2.01, Maastricht University, The Netherlands) was used to determine the exponential curve, pressure, and the internal diameter. At the end of each step, the internal diameter (*μ*m) and the corresponding wall tension (mN/mm^2^) were recorded. When the transmural pressure on the rings reached 100 mmHg, as determined according to the length-tension curves, the stretch-up procedure was stopped and the rings were released to 90% of their internal circumference at 100 mmHg. This degree of passive tension was then maintained throughout the experiment.

### 2.3. Pharmacological Protocols

After normalization, the artery rings were equilibrated for at least 45 minutes.

### 2.4. Relaxation by* Suxiao Jiuxin* Pill in KCl- and U46619-Induced Contraction

Cumulative concentration- (−2~1.25 log mg/mL) relaxation curve for* Suxiao Jiuxin *Pill was established in IMA rings precontracted with K^+^ (KCl 25~40 mM, *n* = 6) or U46619 (10~20 nM, *n* = 6). The concentration of the vasoconstrictors K^+^ [29] or U46619 [38, 39] was determined on the basis of the previous studies. Only one concentration-relaxation curve was obtained from each IMA ring. To allow each concentration of* Suxiao Jiuxin* Pill to reach the relaxing plateau, usually 10~30 min was given before the next dose to be added. Relaxations to* Suxiao Jiuxin* Pill (−2~1.25 log mg/mL) were recorded and expressed as a percentage of the vasoconstrictor-induced precontraction. Glyceryl trinitrate- (GTN) and amlodipine- (both at −10–4.5log⁡⁡*M*) induced relaxations were used in comparison to* Suxiao Jiuxin* Pill.

### 2.5. Depression of Contraction by Pretreatment with* Suxiao Jiuxin* Pill in IMA

After equilibration for at least 60 min, IMA rings were incubated with* Suxiao Jiuxin* Pill (*Suxiao Jiuxin* Pill 1 mg/mL, 10 mg/mL, or vehicle for 30 min). The concentration of* Suxiao Jiuxin* Pill was chosen from the plasma concentration, calculated from the plasma concentration of its major component borneol in the human reported previously [41, 42]. The vehicle ethanol was added in the control group to exclude the effect of ethanol that was used as the solvent of* Suxiao Jiuxin* Pill. In each experiment, one of the two vasoconstrictors was added to construct the cumulative concentration-contraction curve. The cumulative concentration-contraction curve was established for KCl (5~120 mM) and U46619 (−10 ~ −6log⁡⁡*M*), respectively. Only one concentration-contraction curve was obtained from each ring. Data regenerated from our laboratories on the inhibitory effects by GTN- [29] and amlodipine- [43] (both at their own plasma concentrations) induced relaxations were used in comparison to* Suxiao Jiuxin* Pill.

### 2.6. Endothelium-Dependent Relaxation

To investigate the role of endothelium denudation and endothelium-derived NO and eNOS,* Suxiao Jiuxin* Pill- (−2~1.25 log mg/mL) induced relaxation in 40 mM KCl-induced precontraction was examined in endothelium-denuded and eNOS inhibitor L-NNA (300 *μ*M) pretreated IMA rings. The endothelium-intact rings were pretreated with L-NNA for 60 min before adding the KCl. Endothelium-intact IMA rings from the same patient were studied as control.

In the endothelium-denuded IMA rings, the endothelium was removed by gently rubbing the intima with a paper stick [36]. We have repeatedly demonstrated that this method may successfully remove the endothelium and eliminate the endothelium-dependent relaxation. In fact, this method can abolish the acetylcholine-induced endothelium-dependent relaxation as we recently published in the human IMA [44], indicating that the endothelium is successfully removed.

### 2.7. Western Blot Analysis of eNOS

IMA samples were homogenized in lysis buffer (KeyGEN, Inc., Nanjing, China), and the lysates were incubated in ice for 1 hour followed by 10 minutes of centrifugation at 10,000 rpm. After the sample was heated at 100°C for 5 minutes to denature it, 120 mg protein for each sample was separated by 8% polyacrylamide gel electrophoresis (Page Gel, Inc., San Diego, CA) together with the prestained protein ladder (MBI Fermentas, Inc.). The proteins were transferred electrophoretically to the polyvinylidene fluoride membrane (Millipore, Billerica, Mass). The membrane was blocked with blocking buffer (trisbuffered saline solution, 0.1% polysorbate 20, 5% nonfat dry milk) for 3 hours at room temperature and incubated with primary antibody against eNOS (1 : 1000; Cell Signaling Technology, Inc., Boulder, Colo) overnight at 4°C. Equivalent protein on the same lane was confirmed by stripping and reblotting with glyceraldehyde 3-phosphate dehydrogenase (1 : 1000; Cell Signaling Technology). The secondary goat anti-rabbit antibody conjugated to horseradish peroxidase (Santa Cruz Biotechnology, Inc., Santa Cruz, CA) at a dilution of 1 : 5000 was added the next day. Finally, blots were developed with an enhanced chemiluminescence detection system (Amersham Pharmacia ECL reagents; GE Healthcare Biosciences, Piscataway, NJ) and exposed to X-ray films. The protein bands were quantified with QuantityOne software (Bio-Rad Laboratories, Inc., Hercules, CA), normalized by glyceraldehyde 3-phosphate dehydrogenase and expressed as multiples of control. These methods are well used in our laboratories [45, 46].

### 2.8. Data Analysis

The sensitivity of an agent was expressed as EC_50_, the effective concentration that caused 50% of maximal relaxation or contraction. The EC_50_ was determined from each concentration-relaxation curve by a sigmoid logistic curve-fitting equation: *E* = *MA* ^*P*^/(*A* ^*P*^ + *K* ^*P*^), where *E* is response, *M* is maximal relaxation, *A* is concentration, *K* is EC_50_ concentration, and *P* is the slope parameter [39]. A computerized program was used for the curve fitting.

All results were expressed as mean ± SEM. Statistical comparisons of the cumulative responses of relaxation or contraction under different treatments were performed by two-way analysis of variance (ANOVA) with repeated measures (SPSS, Inc., Chicago, IL), which was followed by a post hoc Bonferroni test to detect the individual differences. *P* < 0.05 was considered to be statistically significant.

### 2.9. Materials

Chemicals involved in this study were potassium chloride, U46619 (Cayman Chemical, Ann Arbor, MI), L-NNA (Sigma, St. Louis, MO), and* Suxiao Jiuxin* Pill (Tianjin Zhongxin Pharmaceutical Group Co., Ltd. China).

*Suxiao Jiuxin* Pill was diluted in 75% ethanol to 300 mg/mL and 100 mg/mL and diluted in 30% ethanol to 10 mg/mL. Stock solution of U46619 was kept frozen until required.* Suxiao Jiuxin* Pill was stored at room temperature.

## 3. Results

### 3.1. Resting Parameters of IMA Ring Segments

The internal diameter of the 60 IMA ring segments at an equivalent transmural pressure of 100 mmHg (D100) was 1.87 ± 0.08 cm as determined in the normalization procedure. When the IMA ring segments were set at a resting diameter of 0.9D100, the equivalent transmural pressure was 90.3 ± 0.5 mmHg, and the resting force was 16.1 ± 1.6 mN.

### 3.2. Similar Relaxation Effect of* Suxiao Jiuxin* Pill in KCl- and U46619-Induced Contraction

In the relaxation studies, the precontraction was 16.0 ± 2.3 mN by KCl and 16.6 ± 5.2 mN by U46619 (*P* > 0.05; *n* = 12 in each group).

**Figure 1:**
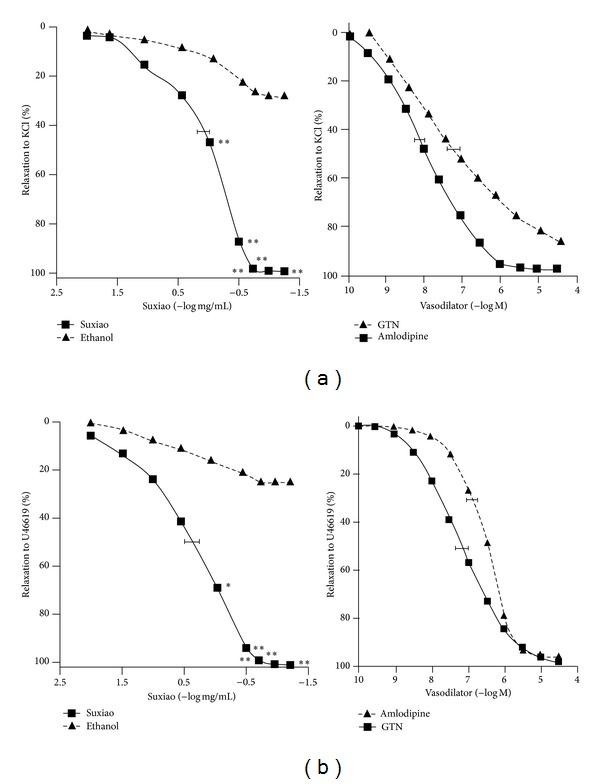
Mean concentration- (log mg/mL for* Suxiao Jiuxin* Pill; −log⁡⁡*M* for GTN and amlodipine) relaxation curves for* Suxiao Jiuxin* Pill, GTN, and amlodipine in KCl- (25~40 mM, (a)) or U46619- (10~20 nM, (b)) induced contraction in human IMA (*n* = 8 in each group). % relaxation to KCl (or U46619): percentage relaxation induced by Suxiao or other vasodilators in the KCl- (or U46619-) induced precontraction. Ethanol was the solvent of* Suxiao Jiuxin* Pill. Values are expressed as mean ± SEM. *P < 0.05, **P < 0.01 compared to vehicle group (unpaired *t*-test). KCl: potassium chloride; GTN: glyceryl trinitrate; Suxiao:* Suxiao Jiuxin* Pill.

*Suxiao Jiuxin* Pill caused full relaxation in KCl- and U46619-precontracted IMA rings (99.4 ± 10.5% for KCl; 99.9 ± 5.6% for U46619; *n* = 6 in each group). Two-way ANOVA for all concentrations revealed that there were no differences (*P* = 0.43; 95% CI: 32.0, 67.8%) between KCl- and U46619-precontraction. The relaxation of* Suxiao Jiuxin* Pill was with similar potency to KCl and U46619 (EC_50_: −0.15 ± 0.23 versus −0.19 ± 0.21 log mg/mL, *P* > 0.05; unpaired *t*-test) (Figure 1). Importantly, two-way ANOVA for all concentrations detected that there were significant differences of relaxation caused by* Suxiao Jiuxin* Pill compared with the control (*P* = 0.002; 95% CI: 29.1, 52.0% for KCl-precontraction and *P* = 0.014; 95% CI: 29.9, 69.8% for U46619-precontraction); there were no differences of relaxation caused by* Suxiao Jiuxin* Pill between KCl- and U46619-precontraction (*P* = 0.43; 95% CI: 22.6, 58.5%).

### 3.3. Depression of Contraction by Pretreatment with* Suxiao Jiuxin* Pill in IMA

**Figure 2:**
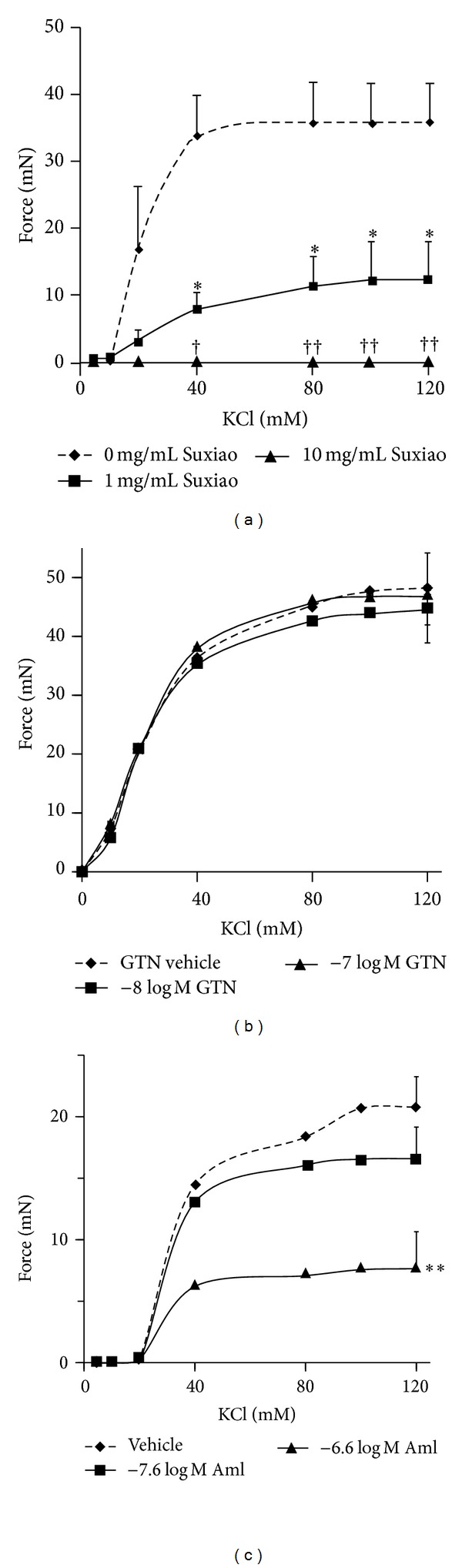
Mean concentration- (mM) contraction (mN) curves for KCl when* Suxiao Jiuxin* Pill 1 mg/mL (■), 10 mg/mL (▲), or  vehicle (◆); GTN −8log⁡⁡*M*(■), −7log⁡⁡*M* (▲), or vehicle (◆); and amlodipine −7.6log⁡⁡*M* (■), −6.6 log M (▲), or vehicle (◆) were added 30 min (except for GTN were added 10 min) before the contraction started in the human IMA ring segments (*n* = 6 in each group). Three rings from an IMA segment taken from the same patient were allocated into each group. Values are expressed as mean ± SEM. *P < 0.05 compared to vehicle group in 1 mg/mL* Suxiao Jiuxin* Pill pretreatment; ^†^ *P* < 0.05, ^††^ *P* < 0.01 compared to vehicle group in 10 mg/mL* Suxiao Jiuxin* Pill pretreatment; **P < 0.01 compared to the control (vehicle) in −6.6log⁡⁡*M* amlodipine pretreatment (unpaired *t*-test). *P* = 0.016 between the treatment of −6.6log⁡⁡*M* amlodipine and the control (vehicle) in KCl-contracted groups. KCl: potassium chloride; GTN: glyceryl trinitrate; Suxiao:* Suxiao Jiuxin* Pill; Aml: amlodipine.

The magnitude of the maximal contraction to KCl was significantly depressed in the group pretreated with plasma concentration of* Suxiao Jiuxin* Pill (1 mg/mL), calculated from the plasma concentration of its major component borneol (*P* = 0.03 to control; 95% CI: 3.1, 43.2 mN; unpaired *t*-test). Two-way ANOVA for all concentrations also demonstrated a significant difference after incubation with 1 mg/mL* Suxiao Jiuxin* Pill compared to the control (*P* = 0.025; 95% CI: −1.9, 16.2 mN). Pretreatment of IMA with* Suxiao Jiuxin* Pill (10 mg/mL) abolished the subsequent contraction to KCl (Figure 2(a)). Figures 2(b) and 2(c) reproduced our previous data on the relaxing effect of the well-studied western vasorelaxant agents glyceryl trinitrate (Figure 2(b)) and amlodipine (Figure 2(c)) at the plasma concentration in comparison to* Suxiao Jiuxin* Pill. In comparison to glyceryl trinitrate and amlodipine, the magnitude of the maximal contraction to KCl was significantly depressed by the plasma concentration of* Suxiao Jiuxin* Pill (1 mg/mL) as mentioned above (Figure 2(a)) but it had no significant change in the plasma concentration of glyceryl trinitrate- (−8log⁡⁡*M*) pretreated (Figure 2(b)) or amlodipine- (−7.6log⁡⁡*M*) pretreated (Figure 2(c)) IMA, although higher concentration of amlodipine (−6.6log⁡⁡*M*) also significantly depressed the maximal contraction of IMA (Figure 2(c)).

**Figure 3:**
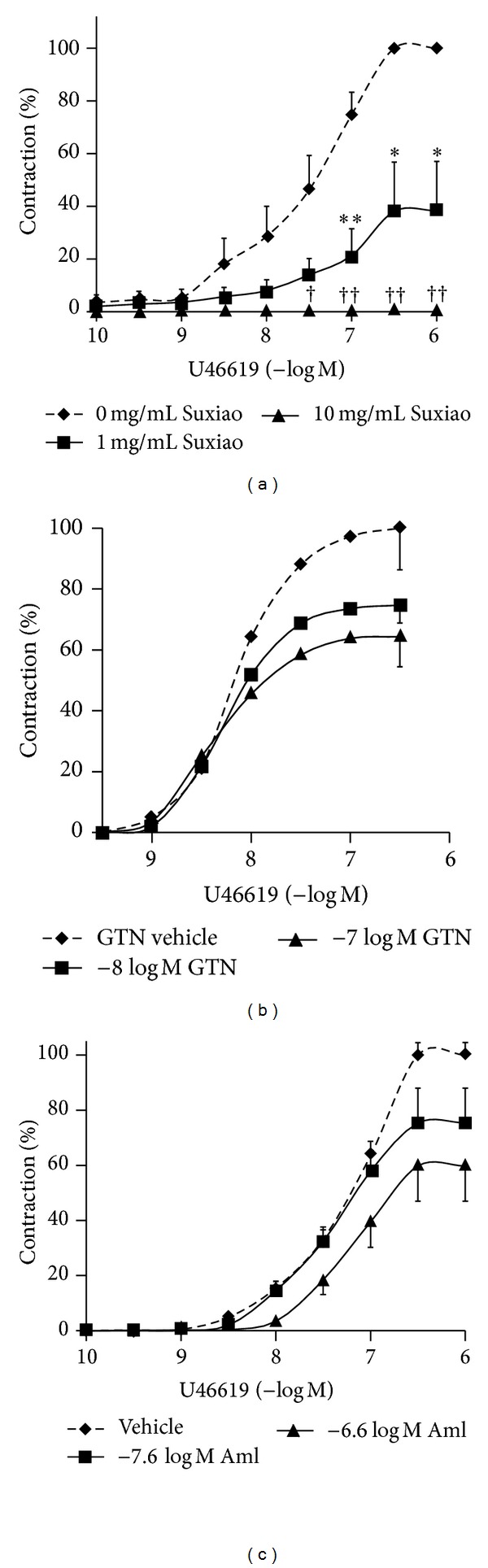
Mean concentration- (−log⁡⁡*M*) contraction (mN) curves for U46619 when* Suxiao Jiuxin* Pill 1 mg/mL (■), 10 mg/mL (▲), or vehicle (◆); GTN −8log⁡⁡*M* (■), −7log⁡⁡*M* (▲), or vehicle (◆); and amlodipine −7.6log⁡⁡*M* (■), −6.6log⁡⁡*M* (▲), or  vehicle (◆) were added 30 min (except for GTN were added 10 min) before the contraction started in the human IMA ring segments (*n* = 6 in each group). Three rings from an IMA segment taken from the same patient were allocated into each group. Values are expressed as mean ± SEM. *P < 0.05 compared to vehicle group in 1 mg/mL* Suxiao Jiuxin* Pill pretreatment; ^†^ *P* < 0.05 compared to vehicle group in 10 mg/mL* Suxiao Jiuxin* Pill pretreatment (unpaired *t*-test). KCl: potassium chloride; GTN: glyceryl trinitrate; Suxiao:* Suxiao Jiuxin* Pill; Aml: amlodipine.

Further, in IMA rings contracted by U46619, incubation with 1 mg/mL* Suxiao Jiuxin* Pill significantly depressed the magnitude of the maximal contraction of U46619 (*P* = 0.007; 95% CI: 4.9; 22.6 mN; unpaired *t*-test). Two-way ANOVA for all concentrations of U46619 demonstrated no significant differences compared to the control (*P* = 0.058; 95% CI: −0.8; 5.1 mN). In addition, pretreatment of IMA with* Suxiao Jiuxin* Pill (10 mg/mL) abolished the subsequent contraction to U46619 (Figure 3). In comparison to the effect of pretreatment with glyceryl trinitrate (Figure 3(b)) and amlodipine (Figure 3(c)), neither the plasma concentration (−8log⁡⁡*M* for glyceryl trinitrate and −7.6log⁡⁡*M* for amlodipine) nor even the 10-fold higher concentrations significantly reduced the maximal contraction to U46619. These data were standardized with percentage of the maximal contraction for easier comparison.

These results show that in comparison to the effect of pretreatment with these three vasorelaxant agents at the plasma concentration, only* Suxiao Jiuxin* Pill caused significant depression effect of the contraction to either KCl or U46619.

### 3.4. At the Concentrations of Less Than 1 mg/mL,* Suxiao Jiuxin* Pill-Induced Relaxation Is Altered by Endothelium-Denudation and L-NNA

**Figure 4:**
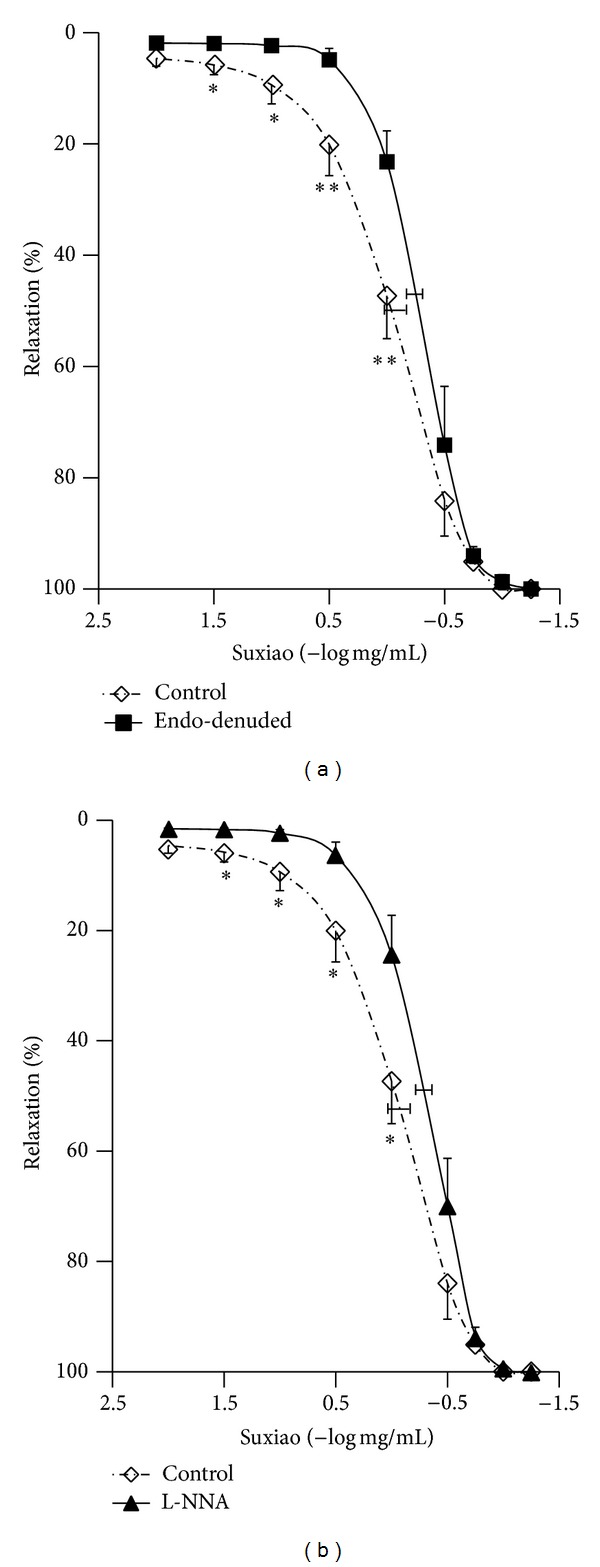
Mean concentration- (log mg/mL) relaxation (%) curves for* Suxiao Jiuxin* Pill (−2~1.25 log mg/mL) in endothelium-denuded (*n* = 6; (a)) and L-NNA (300 *μ*M, *n* = 6; (b)) pretreated IMA rings precontracted with 40 mM KCl. Endothelium-intact IMA rings taken from the same patient were studied as control. Values are expressed as mean ± SEM. *P < 0.05, **P < 0.01, compared with endothelium-intact control group. Suxiao:* Suxiao Jiuxin* Pill; Endo-denuded: endothelium-denuded.

At the concentrations of 31.6 *μ*g/mL, 100 *μ*g/mL, and 316 *μ*g/mL,* Suxiao Jiuxin* Pill-induced relaxation against 40 mM KCl was significantly attenuated by denudation of endothelium (0.3 ± 0.3% versus 4.6 ± 1.8%, *P* < 0.05 for 31.6 *μ*g/mL* Suxiao Jiuxin* Pill; 0.7 ± 0.5% versus 9.3 ± 3.4%, *P* < 0.05 for 100 *μ*g/mL* Suxiao Jiuxin* Pill; 5.4 ± 2.1% versus 24.9 ± 5.6%, *P* < 0.01 for 316 *μ*g/mL* Suxiao Jiuxin* Pill; Figure 4(a)). A similar effect was seen in endothelium-intact IMA rings pretreated with 300 *μ*M L-NNA (0.4 ± 0.3% versus 4.6 ± 1.8%, *P* < 0.05 for 31.6 *μ*g/mL* Suxiao Jiuxin* Pill; 1.3 ± 0.7% versus 9.3 ± 3.4%, *P* < 0.05 for 100 *μ*g/mL* Suxiao Jiuxin* Pill; 8.1 ± 2.4% versus 24.9 ± 5.6%, *P* < 0.05 for 316 *μ*g/mL* Suxiao Jiuxin* Pill; Figure 4(b)).

Taken together, Figure 4 shows that the effect of denudation of the endothelium and addition of NO-inhibitor L-NNA are similar. At the concentrations of less than 1 mg/mL,* Suxiao Jiuxin* Pill-induced relaxation is reduced by both endothelium-denudation and L-NNA. However, the maximal relaxation was not altered. These results show that* Suxiao Jiuxin* Pill-induced relaxation is mainly endothelium-independent although it also has a small endothelium-dependent component.

### 3.5. Western Blot Analysis of eNOS in IMA Pretreated with* Suxiao Jiuxin* Pill

**Figure 5:**
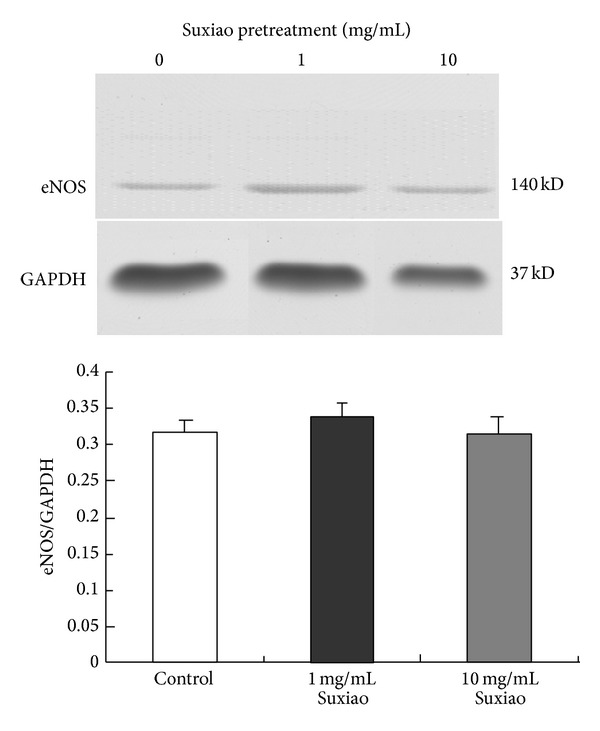
Protein expression of eNOS in human IMA rings. IMA rings were incubated with* Suxiao Jiuxin* Pill 1 mg/mL, 10 mg/mL, or vehicle (Control) for 30 min (*n* = 3). Data are shown as mean ± SEM. Suxiao:* Suxiao Jiuxin* Pill; eNOS: endothelial nitric oxide synthase.

The eNOS expressions in IMA had no significant differences between the control and* Suxiao Jiuxin* Pill (1 mg/mL or 10 mg/mL) pretreated groups (Figure 5).

## 4. Discussion

In this study we have found in the human IMA, the most commonly used arterial graft for coronary artery bypass surgery, that (1) the compound Chinese medicine* Suxiao Jiuxin* Pill had potent vasorelaxant effect on various vasoconstrictor-mediated vasoconstriction, particularly on KCl and U46619; (2)* Suxiao Jiuxin* Pill fully relaxed the contraction caused by KCl and U46619 with similar potencies; (3) pretreatment with* Suxiao Jiuxin* Pill effectively prevented and abolished KCl- or U46619-induced contraction and this effect may be more significant than the effect caused by the pretreatment by GTN and amlodipine; (4) the mechanism of* Suxiao Jiuxin* Pill relaxing human arteries may involve endothelium-dependent mechanism at the concentrations of microgram per milliliter; and (5)* Suxiao Jiuxin* Pill at concentrations of milligram per milliliter presents endothelium-independent relaxation.

With more than 2 decades of clinical application, large amount of clinical pharmacological studies has proved that* Suxiao Jiuxin* Pill has anti-atherosclerosis effect, may improve microenvironment, and may protect myocytes and vascular endothelial cells [47]. Moreover, long-term* Suxiao Jiuxin* Pill intake may also significantly improve heart function and decrease angina pectoris attack as well as protect patients from myocardial infarction [47]. These effects have been obtained not only for emergency lifesaving but also for routine therapy. It is also reported that with the special advantage of no drug tolerance and toxic or side effect, it greatly supplements the deficiency of nitrate esters for daily taking [47]. Owing to the above-mentioned advantages,* Suxiao Jiuxin* Pill is now widely used in the treatment of coronary artery disease.

As mentioned above, the main components of* Suxiao Jiuxin* Pill are tetramethylpyrazine (TMP) and borneol, although there are also a number of other substances contained in this medicine. In recent years, a variety of studies have been performed to investigate the therapeutic effect of TMP, including the vasodilatation and endothelial protection. A study shows that TMP is a dilator of human pulmonary and bronchial arteries through endothelium-independent mechanism and that TMP preferentially relaxes pulmonary resistance vessels rather than large conduit pulmonary arteries [48]. It has also been shown that TMP elicits disparate responses in cardiac contraction and intracellular Ca(2+) transients in isolated adult rat ventricular myocytes [12]. TMP in treating human arteriosclerosis obliterans not only displays extraordinary effect, but also has good effect on curing the damage of endothelial cells [49]. Moreover, TMP has been shown to have protective effects on the vascular endothelial function in patients undergoing PCI [50] and on H_2_O_2_-induced oxidative damage in human umbilical vein endothelial cells due to its antioxidant and antiapoptotic properties by downregulating nitric oxide (NO) and nitric oxide synthase (NOS) production [49] and by scavenging ROS and regulating intracellular calcium concentration [51]. In addition, TMP at micromolar concentrations stimulated NO production in human platelets via a novel mechanism that activated eNOS protein expression [52] and produced a concentration-dependent relaxation in the aortic rings precontracted with vasopressin or phenylephrine that is related to the opening of SK_Ca_ and K_ATP_ channels [53].

In comparison to the well-studied component TMP, the role of borneol in* Suxiao Jiuxin* Pill is less clear but this component is frequently used in traditional Chinese medicine [54]. There is lack of studies on the effect of borneol on vascular tone. However, borneol may have benefits for the neuroprotective effect of sodium ferulate against injury induced in the brain by ischaemia/reperfusion [55]. More likely, borneol may promote the absorption of other components in the compound Chinese medicine as it has been shown that borneol promoted nasal absorption of Ligustrazine into brain [56].

On the basis of the above findings, we explored the vasorelaxation of* Suxiao Jiuxin* Pill and possible mechanisms in the human IMA in the present study. We demonstrated that* Suxiao Jiuxin* Pill relaxed all IMA segments against various vasoconstrictors representing different vasoconstriction mechanisms, including KCl and U46619. KCl was used to depolarize the smooth muscle cells to open the voltage-operated channel (VOC) [57], whereas U46619 may bind the receptors to activate the receptor-operated channel (ROC) [29]. The results show that* Suxiao Jiuxin* Pill has full relaxant effects on contractions induced by both vasoconstrictors with similar potencies, indicating a high effectiveness of this vasodilator agent (Figure 1(a)). In comparison, in our previous study, the calcium antagonist amlodipine induced full relaxation in contractions mediated by these two vasoconstrictors but the potency was higher in the vasoconstriction mediated by KCl than that mediated by U46619 [43]. In contrast,* Suxiao Jiuxin* Pill rapidly relaxed the IMA regardless of the nature of the constrictor stimulus. Further, we previously demonstrated that GTN could fully relax the IMA precontracted by KCl or U46619 with similar EC_50_ values [29]. On this point,* Suxiao Jiuxin* Pill is similar to GTN with no significant constrictor selectivity in the relaxation.

Importantly,* Suxiao Jiuxin* Pill had significant effect on inhibition if used prior to contraction. Indeed, pretreatment of* Suxiao Jiuxin* Pill at 1 mg/mL significantly depressed the contraction mediated by both KCl and U46619 and at 10 mg/mL, the contraction was even abolished (Figures 2(a) and 3(a)). This effect was significantly higher than that by the plasma concentration of either GTN or amlodipine (compare Figures 2(a)–2(c) and 3(a)–3(c)). Amlodipine is known as a calcium antagonist [58]. The clinical implication of this result is strong. This implies that at the plasma concentration, the antispastic effect (prevention of strong contraction) of* Suxiao Jiuxin* Pill is significantly higher than that of either GTN or amlodipine at the plasma concentration of these vasodilators. Such effect may be favorable for the patients who have angina pectoris or the patients who have had CABG operation with IMA grafting for prophylactic therapy of vasospasm. Our findings are in accordance with the recent report demonstrating that* Suxiao Jiuxin* Pill could improve the pre- and post-PCI coronary artery flow rate, increase the collateral artery patency, and reduce the incidence of perioperative myocardial infarction of acute coronary syndrome patients [59].

The present study clearly shows that* Suxiao Jiuxin* Pill-induced relaxation in the human artery has two components—endothelium-dependent and -independent mechanisms. The endothelium-dependent mechanism is the major component of the relaxation; the endothelium-dependent effect exists but it is a small effect. At microgram per milliliter concentrations the relaxation is endothelium-dependent, as shown in Figure 4. Indeed, either endothelium-denudation or L-NNA inhibited the* Suxiao Jiuxin* Pill-induced relaxation at the lower concentrations. At higher concentrations (higher than 1 mg/mL), however, the role of endothelium became insignificant. The endothelium-independent relaxation was obviously due to the direct relaxing effect of* Suxiao Jiuxin* Pill on the smooth muscle. Interestingly, western blot analysis showed that* Suxiao Jiuxin* Pill had no effect on the protein expression of eNOS (Figure 5). Taken together with the result that the eNOS inhibitor L-NNA had inhibitory effect on the relaxation, the present study suggests that the endothelium-dependent component at lower concentrations of* Suxiao Jiuxin* Pill is most likely associated with posttranslational modification of eNOS, which, however, warrants further investigation.

In conclusion, the present study suggests that* Suxiao Jiuxin* Pill has a rapid and potent vasorelaxant effect on human IMA precontracted by a variety of vasoconstrictors with similar potency. Pretreatment with* Suxiao Jiuxin* Pill has high potency in inhibiting contraction induced by membrane depolarization and receptor (thromboxane) mechanisms. The vasorelaxation induced by* Suxiao Jiuxin* Pill involves both endothelium-dependent and -independent mechanisms. The present study demonstrates a full and effective vasorelaxation effect of* Suxiao Jiuxin* Pill and supports the use of this widely used medication in coronary artery disease including CABG patients, in favor of treating and preventing graft spasm. Therefore, the effect of* Suxiao Jiuxin* Pill on human arteries demonstrated in this study may prove to be particularly important in vasorelaxing therapy in cardiovascular disease.
